# Temporal Stability of Vagally‐Mediated Heart Rate Variability Over a One‐Year Period: A Latent State–Trait Examination

**DOI:** 10.1111/psyp.70445

**Published:** 2026-11-05

**Authors:** Magdalena K. Wekenborg, Clemens Kirschbaum, LaBarron K. Hill, R. Arne Wittling, Julian Eder, Lorenzo Filosa, Julian F. Thayer

**Affiliations:** ^1^ Else Kröner Fresenius Center for Digital Health, Faculty of Medicine and University Hospital Carl Gustav Carus TUD Dresden University of Technology Dresden Germany; ^2^ Dresden LABservice GmbH Dresden Germany; ^3^ Department of Psychology North Carolina Agricultural & Technical State University Greensboro North Carolina USA; ^4^ ZNF – Center for Neuroscience Research NPO Trier Germany; ^5^ Department of Psychology, Faculty of Medicine and Psychology Sapienza University of Rome Rome Italy; ^6^ Department of Psychology University of California, Irvine Irvine California USA

**Keywords:** biomarker, cardiac vagal tone, heart rate variability, latent state–trait modeling, temporal stability

## Abstract

Vagally‐mediated heart rate variability (vmHRV) is widely proposed as a biomarker for physical and mental health, but its utility hinges on psychometric robustness, particularly temporal stability and consistency across measurement contexts. Prior reliability studies have been constrained by small samples, short retest intervals, and analytic approaches that cannot disentangle stable individual differences from situational fluctuations and measurement error. The present study examined the temporal and transsituational stability of vmHRV over a 1‐year interval in a large, population‐based sample (*N* = 175) from the Dresden Burnout Study. Short‐term inter‐beat‐interval (IBI) recordings were obtained at two annual assessments under two conditions: recumbent recovery following venipuncture and seated rest. Four cardiac and HRV‐derived parameters, the established vmHRV indices root mean square of successive differences (RMSSD), percentage of successive RR intervals differing by more than 50 ms (pNN50), and high‐frequency power (HF power), complemented by mean RR interval (RR) as a general cardiac timing parameter, were analyzed using single‐trait–multistate–method‐minus‐one [STMSM(M–1)] models within the latent state–trait (LST) framework, with recovery specified as the reference condition and resting modeled as a condition‐specific method factor. Reliability ranged from 0.650 to 0.903, and trait specificity ranged from 0.375 to 0.773. RR and RMSSD showed the most consistent trait‐like profiles across occasions and conditions. pNN50 showed the highest individual trait‐specificity estimate, but also greater heterogeneity across occasions, whereas HF power showed comparatively lower trait specificity and greater occasion‐specific variance. Method specificity was small (0.034–0.087), indicating that stable condition‐specific deviations from recovery accounted for a limited proportion of variance. These findings support the interpretation of vmHRV, particularly RMSSD and pNN50, as mainly trait‐like indicators of cardiac vagal function, while also showing that single short‐term assessments contain meaningful occasion‐related variance. Standardized assessment conditions and repeated measurements remain essential for biomarker applications of vmHRV.

## Impact Statement

Heart rate variability is increasingly used as a health biomarker, yet its psychometric robustness over longer periods remains unclear. Using latent state–trait modeling in a large population‐based sample assessed 1 year apart, this study shows that vagally‐mediated indices reflect largely stable individual differences, while also carrying meaningful occasion‐specific variance. The findings clarify which cardiac measures are trait‐like and underscore why standardized conditions and repeated assessments matter for biomarker use.

## 1 Introduction

Besides the initial focus on sympathetic nervous system (SNS) activity, there is a growing literature on the role of parasympathetic activity in the maintenance of health. This influence is mediated primarily by the vagus nerve, the main efferent pathway of the parasympathetic nervous system (PNS). Increased cardiac vagal activity has been linked to cognitive, emotional, and behavioral flexibility, supporting adaptive adjustment to environmental demands (Appelhans and Luecken 2006; Grol and De Raedt 2020; Hildebrandt et al. 2016; Thayer et al. 2012; Thayer and Koenig 2025). In contrast, chronically reduced cardiac vagal activity has been associated with impaired emotional regulation and a range of somatic and psychological pathologies (Azulay et al. 2022; Cheng et al. 2022; Gerritsen et al. 2001; Jandackova et al. 2016; Kemp et al. 2010; Ramesh et al. 2023; Thayer et al. 2010; Wekenborg et al. 2022; Williams et al. 2019). The most widely used and currently best validated marker of cardiac vagal activity is vagally‐mediated heart rate variability (vmHRV), defined as high‐frequency variations in the interval length between consecutive heart beats. HRV more broadly arises from the interplay of the sympathetic and the parasympathetic branches of the autonomic nervous system (ANS) at the sinoatrial node of the heart. Because PNS influences can modulate heart rate on the millisecond timescale, whereas SNS influence operates more slowly, vmHRV provides a relatively pure index of vagal function (Lewis et al. 2012; Saul 1990; Task Force of the European Society of Cardiology and the North American Society of Pacing and Electrophysiology 1996). Keeping in mind the numerous empirical findings on the ability of vmHRV to predict psychopathology (Jandackova et al. 2016; Wekenborg et al. 2022), as well as morbidity and mortality especially with respect to cardiovascular pathologies (Fang et al. 2020; Gerritsen et al. 2001; Jarczok et al. 2022; Lee et al. 2023; Thayer et al. 2010), it seems reasonable to suggest vmHRV as a potential biomarker for mental and physical health. However, in order to use vmHRV as a reliable biomarker, it is essential to establish its physiometric robustness (Gloger et al. 2020), particularly its temporal stability and reliability across different measurement contexts.

There is already a substantial body of work examining the reliability of vmHRV (Bertsch et al. 2012; Dragomir et al. 2014; Kleiger et al. 1991; Pinna et al. 2007; Schroeder et al. 2004; Schumann et al. 2025; Seidman et al. 2024; Sinnreich et al. 1998). Most studies have focused on operationalizing vmHRV through the root mean square of successive differences between adjacent interbeat intervals (RMSSD), and the spectral power in the high‐frequency band (0.15–0.40 Hz) that reflects parasympathetic modulation of heart rate (HF‐HRV). Occasionally, other indices such as the percentage of successive interbeat intervals differing by more than 50 ms (pNN50) have also been used.

Reliability has typically been quantified using intraclass correlations (ICCs), which indicate the proportion of variance in repeated measurements that is attributable to stable between‐person differences rather than within‐person fluctuations. Other approaches have also been applied, such as simple test–retest correlations or more complex coefficients of reliability that estimate the variance proportion explained by trait components. Across studies, values generally indicate moderate to high reliability, with ICCs ranging from 0.32 to 0.91 (most around 0.70) and correlation coefficients between 0.69 and 0.73.

However, existing studies differ widely in sample size (14 to 134 participants) and follow‐up duration, with many relying on relatively small samples and short intervals of a few hours to 1 week (Bertsch et al. 2012; Besson et al. 2025; Guijt et al. 2007; Pinna et al. 2007; Schumann et al. 2025), several weeks (Borges et al. 2018; Kleiger et al. 1991; Schroeder et al. 2004), or a few months (Pitzalis et al. 1996; Sinnreich et al. 1998; Uhlig et al. 2020). The few existing studies on vmHRV with longer time intervals (1–6 years), which are particularly relevant for the use of vmHRV as a biomarker, indeed reveal moderate to high stability, with some evidence suggesting that stability may decrease as the interval lengthens (Burleson et al. 2003; Goedhart et al. 2007; Hu et al. 2017; Li et al. 2009; Seidman et al. 2024). However, the limited evidence on long‐term stability beyond 1 year is difficult to interpret, as most of these studies were based on very small samples with fewer than 20 participants and often relied on selective participant inclusion. For biomarkers to be clinically meaningful predictors, however, it is crucial to demonstrate robust stability across longer time spans and in larger, more diverse populations. Beyond temporal stability, transsituational consistency is also critical: Evidence that the stability of vmHRV varies depending on the measurement condition (Bertsch et al. 2012; Burleson et al. 2003; Li et al. 2009; Salomon 2005; Sloan et al. 1995) highlights the need for systematic investigation of reliability across different contexts, too.

Another challenge lies in the analytic strategies commonly employed. Commonly used indices such as ICCs, test–retest correlations, or coefficients of variation provide useful first insights but cannot disentangle stable interindividual differences (traits) from situational variability (states) and measurement error. As a result, trait‐like consistency may be biased if not rightly disentangled from state‐like components.

In summary, there is evidence of a moderate to high stability of vmHRV. However, given that some studies reported medium to low reliability (Borges et al. 2018; Pitzalis et al. 1996), together with the methodological limitations (e.g., small selective samples, short test–retest intervals, drawbacks of commonly used statistical analyses), firm conclusions on the reliability of vmHRV would be premature.

To address these limitations, the present study investigates the re‐test reliability of short‐term vmHRV recordings over a 1‐year period in a large, population‐based sample during two different measurement conditions: a sitting resting condition and a lying recovery condition, using different time‐ and frequency‐domain cardiac and HRV‐derived parameters, employing a latent state–trait (LST) modeling framework (Steyer et al. 1992, 2015; Steyer and Schmitt 1990). LST models, grounded in structural equation modeling (SEM), go beyond classical test theory and offer a powerful and flexible approach for partitioning the total variance of repeated measures into three distinct sources: (1) trait variance, representing stable individual differences that persist across time and situations; (2) state variance, capturing occasion‐specific deviations that reflect context‐sensitive fluctuations or temporary conditions; and (3) error variance, reflecting unreliability or measurement noise. By explicitly modeling these components, LST analyses allow for a more precise estimation of the consistency of a construct over time and across measurement conditions, key requirements for considering vmHRV as a reliable biomarker of health. Unlike conventional reliability estimates, LST models do not assume that stability equals reliability. Instead, they test this assumption by decomposing the observed data structure. This methodology is especially appropriate in the study of vmHRV, which is known to reflect both enduring physiological capacities (i.e., tonic vagal activity) and dynamic responsiveness to contextual demands (i.e., phasic vagal activity). By modeling vmHRV data collected under two distinct but relevant conditions at two separate time points spaced 1 year apart, the present study leverages the strengths of LST modeling to examine both the temporal and transsituational stability of vmHRV. Previous research has demonstrated the utility of LST models in psychophysiology and affective neuroscience, where they have been successfully applied to evaluate the trait‐like consistency of individual differences in autonomic and hormonal markers (Bertsch et al. 2012; Hagemann et al. 2005; Hellhammer et al. 2007). Importantly, Bertsch et al. (2012) were the first to apply LST modeling specifically to vmHRV data. In their study, they examined the short‐term stability of vagally mediated HRV indices across multiple sessions (i.e., three sessions, 1 week apart) and conditions (i.e., spontaneous breathing, paced breathing, and reaction time task) in a sample of 60 university students.

Their findings confirmed that a substantial proportion of variance in RMSSD, pNN50, and HF power reflects stable individual differences, while also highlighting the presence of context‐ and occasion‐specific fluctuations.

Building on previous evidence, the present study applies LST modeling to vmHRV data in order to move beyond average‐level comparisons and bivariate correlations, offering a more nuanced and psychometrically robust understanding of vmHRV as a potential biomarker across time and conditions. Extending prior work, our study introduces several key innovations. First, we employed a larger, more heterogeneous, population‐based sample and used a considerably longer retest interval (1 year), enabling the assessment of long‐term temporal stability. Second, we implemented a condition‐specific method factor within the modeling framework, treating recovery as the reference method and modeling resting as a structured deviation. This approach allowed for a refined decomposition of trait, state, and method variance, offering greater insight into condition‐specific influences on vmHRV. Third, whereas previous LST work on HRV (Bertsch et al. 2012) focused exclusively on vagally mediated indices (RMSSD, pNN50, and HF power), we additionally examined a general cardiac timing parameter (mean RR interval). This allowed us to test whether trait‐like stability is specific to vagally mediated measures or generalizes to other cardiac and HRV‐derived parameters.

As no universally accepted cutoffs exist for LST coefficients, we interpreted them in relation to each other and to previous findings. A parameter was considered predominantly trait‐like when trait specificity exceeded 0.50, that is, when stable between‐person differences accounted for the majority of observed variance and clearly exceeded occasion specificity. Estimates were further compared with those of Bertsch et al. (2012), the only previous LST study of vmHRV. Conventional ICCs were interpreted following Koo and Li (2016), with values below 0.50 indicating poor, 0.50 to 0.75 moderate, 0.75 to 0.90 good, and above 0.90 excellent reliability.

Through this design, we aimed to provide a more ecologically valid and comprehensive evaluation of the trait‐like properties of vmHRV and its potential as a reliable individual difference marker.

## 2 Materials and Methods

### 2.1 Participants

The present sample is part of the ongoing prospective Dresden Burnout Study (DBS), a large‐scale longitudinal study designed to assess psychological, societal, and biological risk factors of burnout systematically (Penz et al. 2018). DBS participants had been recruited Germany‐wide through newspapers, social media, and the civil register of the city of Dresden. An age between 18 and 68 years and German language skills were selected as the only inclusion criteria to accrue a large professionally and socioeconomically diverse sample. Participants were not selected on the basis of any psychiatric or somatic diagnosis; the sample therefore reflects the range of health characteristics present in the general working‐age population.

The data used in the present study were obtained during an annual in‐person biomarker assessment, to which all DBS participants with residence in Dresden and within a 60 km radius around the city are invited. At T1 (September to October 2015), 446 participants accepted our invitation to participate. At T2 (October 2016 to February 2017), 507 participants accepted our invitation. Altogether, a total of 175 individuals (120 women, *M* age_T1_ = 42.15 years, SD_T1_ = 11.52, range_T1_ 21–67 years) provided HR recordings and complete data on all relevant socio‐demographic variables at both measurement time points. Sample characteristics are depicted in Table 1. Participant sex was recorded as a binary self‐report (male/female). Sex assigned at birth and gender identity were not assessed separately.

**TABLE 1:** Demographic and health‐related characteristics of the sample at the first visit (T1) and follow‐up (T2).

	T1		T2	
	*M* (SD)	Range	*M* (SD)	Range
Demographics				
Age (years)	42.15 (11.52)	21–67	43.49 (11.35)	23–68
Male (%)	31.4			
BMI (kg/m^2^)	24.62 (4.16)	17.30–39.14	24.66 (4.29)	19.06–38.46
Health related factors				
Smokers (%)	11.6		9.8	
Caffeine consumer (%)	93.0		93.1	
Alcohol consumer (%)	86.0		89.7	

Abbreviation: BMI, body mass index.

All participants gave written informed consent and received a monetary reward of 15€ at T1 and T2. The study protocol was approved by the ethics committee of TUD Dresden University of Technology (EK236062014) and was conducted in accordance with the Declaration of Helsinki.

### 2.2 Procedure

The full biomarker sampling procedure lasted approximately 50 min and was conducted between 08:00 and 19:00 h.

One week before the biomarker sampling, participants completed an online questionnaire assessing demographic and health‐related factors.

After arriving in the laboratory, participants signed the consent forms and were provided with heart rate devices. They were then brought to the blood sampling room, where they lay down on a stretcher. HRV was recorded during the blood draw and participants remained in a supine position for 6 min, during which recordings of recumbent recovery HRV were obtained. Approximately 20 min after completion of the recovery, participants were led to a separate room, where 6 min of seated resting HRV was assessed. During this period, participants were instructed to sit quietly with their eyes open.

Based on both previous evidence showing that venipuncture induces psychological and physiological stress responses (Weckesser et al. 2014) as well as significant differences between HR during blood sampling and recovery which we reported previously (Kanthak et al. 2017), venipuncture was considered an emotionally‐arousing situation and the subsequent post‐venipuncture time period as a recovery period. Throughout this study, recovery refers to absolute levels of cardiac vagal indices during this post‐venipuncture supine period. No difference scores were computed, as the focus was on the stability of vagal levels rather than on reactivity or recovery capacity.

### 2.3 Data Recording and Quantification

Millisecond‐based inter‐beat interval (IBI) data were recorded with a Polar RS800 CX system via corresponding chest belt (Polar Electro OY, Kempele, Finland) during the whole biomarker sampling procedure at a 1‐kHz sampling rate. Of the complete IBI timeline, the following data were used: (1) a 335‐s recumbent recovery interval directly after the venipuncture, (2) a 335‐s seated resting condition approximately 20 min after completion of the recovery period. An interval after the venipuncture was chosen to avoid distortion of resting HRV by anticipatory fears. The interval during venipuncture was not included as it lasted only 90 s.

The data were then transferred to the Polar Precision Performance Software (Polar Electro OY, Kempele, Finland) and exported as the raw IBI data for further analysis. The IBI data were automatically checked for artifacts and corrected by the Center for Neuroscience Research NPO (Trier, Germany) using the NEUROCOR precision HRV algorithm (Wittling 2014). The preprocessing and computation of HRV indices followed the recommendations of the Task Force of the European Society of Cardiology and the North American Society of Pacing and Electrophysiology (1996), as implemented in the NEUROCOR analysis process. The algorithm marked all IBIs outside of age‐dependent physiological limits. All intervals where the moving average over five consecutive IBIs exceeded an age‐dependent threshold were marked and replaced by spline‐interpolated IBIs of equivalent length without altering the total duration of the signal. In cases of extended artifact phases, the distribution of R‐wave timings was adjusted to match the R‐wave dynamics before and after the artifact. The percentage value of the artifact load, based on the total number of IBIs and the number of corrected IBIs, was calculated. Phases with an artifact load > 5% were excluded. Prior to frequency‐domain analysis, IBI series were DC‐corrected using a parabolic curve‐fitting procedure to remove slow baseline trends. Given the standardized short‐term recording conditions, stationarity requirements for FFT‐based spectral analysis were assumed to be sufficiently met by the study design; therefore, higher‐order detrending procedures such as cubic or smoothing splines were not applied in order to avoid unnecessary removal of physiologically relevant variability.

The following four cardiac and HRV‐derived parameters were used for statistical analyses: (1) root mean square of successive differences (RMSSD), (2) percentage of consecutive RR intervals that differ by more than 50 ms (pNN50), (3) power in the high frequency band (HF power), and (4) mean inter‐beat interval in milliseconds (RR).

RMSSD, pNN50, and HF power were considered established indices of vagally mediated HRV and parasympathetic cardiac modulation (Laborde et al. 2017; Task Force of the European Society of Cardiology and the North American Society of Pacing and Electrophysiology 1996). Mean RR interval was included as a general cardiac timing parameter that may capture stable interindividual differences in cardiac regulation.

### 2.4 Statistical Analyses

#### 2.4.1 Conventional Test–Retest Analyses

As an initial descriptive index of temporal stability, zero‐order Pearson correlations between T1 and T2 were calculated separately for each cardiac and HRV‐derived parameter and measurement condition. These correlations quantify the extent to which participants maintained their relative rank ordering across the one‐year interval and were considered descriptive complements to, rather than substitutes for, the subsequent ICC and LST analyses.

To complement the LST variance decomposition with conventional indices of one‐year test–retest reproducibility, intraclass correlation coefficients (ICCs) were additionally estimated separately for each cardiac and HRV‐derived parameter and measurement condition. Following recommendations for test–retest reliability (Koo and Li 2016), we used two‐way mixed‐effects, single‐measurement models and estimated both consistency and absolute‐agreement ICCs, together with 95% confidence intervals. The consistency definition evaluates preservation of between‐person differences while disregarding systematic differences between measurement occasions, whereas the absolute‐agreement definition additionally incorporates such systematic differences into the error component. We therefore report both coefficients to distinguish these complementary aspects of test–retest reproducibility (De Vet et al. 2006; Koo and Li 2016). These conventional coefficients were considered complementary to, rather than interchangeable with, the LST coefficients: in the LST framework, reliability comprises all error‐free latent variance, whereas trait specificity quantifies the proportion attributable specifically to stable trait‐related individual differences. Correlations and ICCs were computed using all available T1–T2 pairs for each parameter and condition (pairwise deletion).

#### 2.4.2 SEM Analyses

Latent state–trait (LST; Steyer et al. 1992, 2015) models were estimated within a structural equation modeling (SEM) framework to decompose the variance of each cardiac and HRV‐derived parameter into components reflecting stable individual differences, occasion‐specific fluctuations, condition‐specific method variance, and measurement error. In contrast to conventional test–retest correlations or intraclass correlations, LST models explicitly distinguish the proportion of observed variance attributable to a stable latent trait from variance due to transient situational influences and unreliability (Steyer et al. 2015). This framework was therefore particularly appropriate for the present study, in which each parameter was assessed repeatedly across two measurement occasions and two physiological conditions, as they have already been applied to assess transsituational consistency of potential biomarkers (Hagemann et al. 2002; Hellhammer et al. 2007; Hermes et al. 2009).

Because the analyses drew on an existing longitudinal cohort, sample size was not determined a priori. The available *N* = 175 nevertheless yielded observations‐to‐parameter ratios well above the recommended minimum (≥ 10:1) and was considerably larger than in prior latent state–trait studies of vmHRV (Bertsch et al. 2012), supporting its adequacy for the present models.

For each cardiac and HRV‐derived parameter, a separate single‐trait–multistate–method‐minus‐one model [STMSM(M–1)] was fitted. The four parameters were RMSSD, pNN50, HF power, and mean RR interval. In each model, the four manifest indicators corresponded to the parameter measured during recovery and resting at T1 and T2.

The STMSM(M–1) specification followed the logic of LST models with method effects, in which one method is selected as the reference and the remaining method‐specific variance is modeled relative to that reference method (Geiser and Lockhart 2012). Usually, in LST with method factors, one condition (in this case, recovery) is designated as the reference method. Recovery was chosen as the reference because the recumbent post‐venipuncture interval represents a physiologically standardized supine state capturing parasympathetic reactivation toward baseline (Laborde et al. 2017). Modeling seated rest as a method factor relative to recovery thus isolates systematic deviations associated with postural and contextual differences between the two conditions. The remaining method (resting) is modeled as a method factor that captures systematic deviations in the respective parameter from the reference condition after accounting for trait/state influences.

Importantly, the method factor isolates situation‐specific variance that is not explained by the general trait‐like or the occasion state variance of the respective parameter measured under the reference condition (Geiser and Lockhart 2012). Thus, the model allows the simultaneous estimation of trait, state, and method effects while controlling for measurement error.

The inclusion of a method factor, and its definition as a residual variance component orthogonal (i.e., not correlated) to the trait factor, is crucial for disentangling whether differences across measurement conditions reflect true psychological states (e.g., autonomic shifts due to context) or are artifacts of the method itself. This is especially relevant for vmHRV, where even small contextual differences (e.g., lying down vs. sitting, anticipation vs. relaxation) can introduce structured variance not attributable to dispositional traits. By modeling these method effects explicitly, we gain a more accurate decomposition of the total variance and avoid conflating state‐like variability with measurement unreliability or stable trait characteristics.

As can be seen in Figure 1, all factor loadings were fixed to one, yielding a parallel‐measurement specification. Within this model, each manifest score was decomposed into an error‐free latent state component and a measurement error component. The latent state components at T1 and T2 were then decomposed into a common latent trait factor and occasion‐specific state‐residuals. The latent trait factor represents stable individual differences that are consistent across both time points and measurement conditions. The state‐residuals (State 1 and State 2) represent occasion‐specific deviations from the trait level, including effects of the measurement occasion and person‐by‐occasion interactions.

**FIGURE 1:**
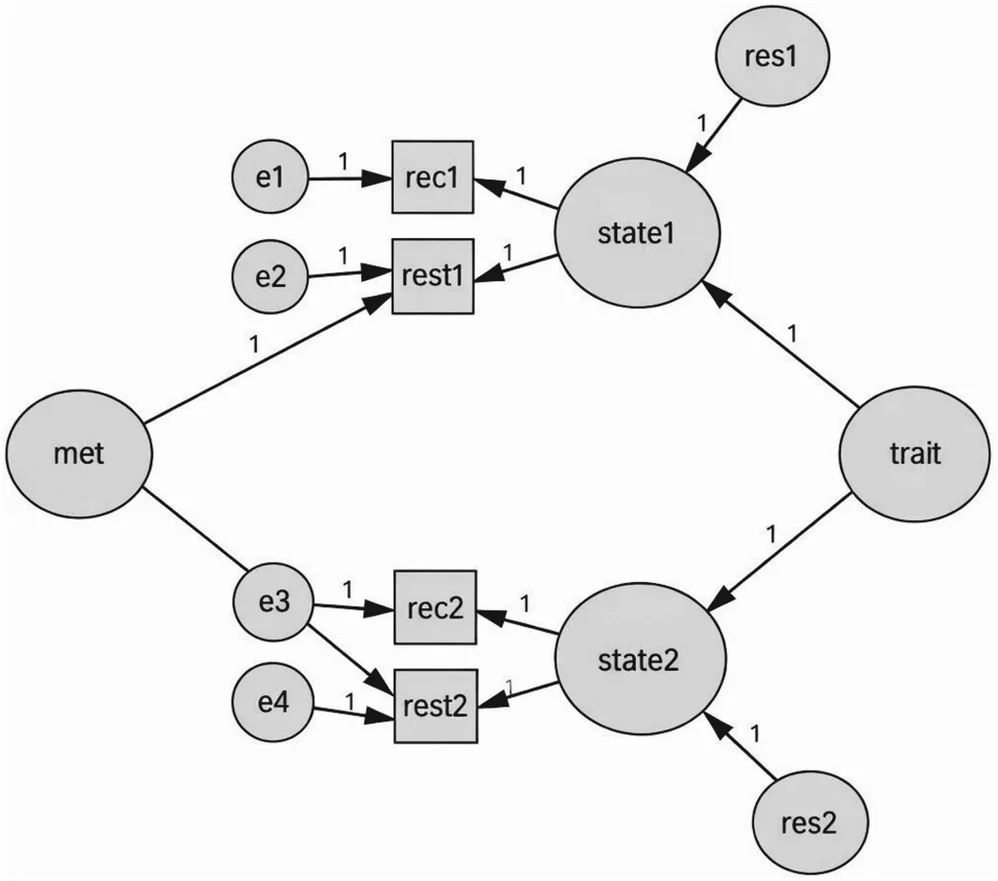
Latent state–trait model for two measurement time points and two measurement conditions (resting and recovery). rec_i_ and rest_i_ are the manifest variables (the respective cardiac or HRV‐derived parameter). e_*i*_ are latent errors. state_*i*_ are latent states. trait is the latent trait. res_*i*_ are latent state‐residuals. met is the method factor representing the measurement conditions.

The resting method factor (met) represents stable condition‐specific variance in the resting indicators that is not shared with the recovery reference method. Measurement error terms (e^1^‐e^4^) represent variance unique to each manifest indicator that is not explained by the latent trait, state‐residuals, or method factor.

Because recovery served as the reference method, no method factor was estimated for recovery indicators. Accordingly, for recovery, trait specificity corresponds to the proportion of variance explained by the common latent trait. For resting, two trait‐related coefficients were distinguished: common trait specificity, reflecting the variance shared with the recovery‐reference trait, and total trait specificity, reflecting the sum of common trait specificity and resting‐specific method variance.

This distinction is important in M–1 models because stable method‐specific variance in the non‐reference condition is treated as a reliable component of the observed score, but one that is not shared with the reference condition (Geiser and Lockhart 2012).

Regarding model identification, we highlight that the present specification is a constrained variance‐components model and should not be interpreted according to the conventional heuristic that every latent factor requires at least three freely loading manifest indicators (Steyer et al. 1992, 2015). In multistate–singletrait LST models, identification results from the complete set of restrictions imposed on the observed covariance structure. In particular, the common trait is shared across occasions, occasion‐specific state‐residuals are orthogonal to the common trait and to one another, measurement errors are independent across occasions and from the latent state components, and the structural loadings used in the present decomposition are fixed to one. These restrictions are consistent with the assumptions of multistate–singletrait LST models described by Steyer et al. (2015), who explicitly demonstrate that trait and state‐residual variances can be identified with two observed variables at each of two measurement occasions. In the present STMSM(M−1) extension, the resting‐specific method factor additionally represents stable variance in the non‐reference condition that is orthogonal to the common trait and occasion‐specific residual components. Accordingly, the four observed scores, recovery and resting at T1 and T2, provide information about distinct stable, occasion‐specific, method‐specific, and residual variance components through their pattern of variances and covariances. All models were overidentified (M0: df = 6; M1: df = 3; M2: df = 2).

Models were estimated using maximum likelihood estimation with 1000 bootstrap replications for standard errors.

Full Information Maximum Likelihood (FIML) method was applied to account for missing observations, since we also included some participants with incomplete data. Separate models were estimated for each parameter, following a procedure previously introduced by Hagemann et al. (2002). Therefore, for each parameter, a sequence of increasingly less restrictive nested models was tested. The initial model, M0, constrained all four error variances to equality and constrained the two state‐residual variances to equality. Model M1 freely estimated the error variances while retaining equality constraints on the two state‐residual variances. Model M2 freely estimated both the error variances and the two state‐residual variances. This stepwise model‐building strategy allowed us to evaluate whether relaxing equality constraints improved model fit sufficiently to justify the additional complexity.

Model fit was evaluated using the chi‐square test statistic, comparative fit index (CFI), Tucker–Lewis index (TLI), root mean square error of approximation (RMSEA), standardized root mean square residual (SRMR), Akaike information criterion (AIC), and Bayesian information criterion (BIC). We accepted CFI values > 0.90 and RMSEA values < 0.08 as indicators of adequate fit (Kline 2016). Nested models were compared using chi‐square difference tests and changes in CFI (ΔCFI; we considered a meaningful change in CFI when ΔCFI ≥ 0.010; Cheung and Rensvold 2002), while also considering absolute fit, information criteria, and parsimony. When the more restrictive model showed acceptable fit and the relaxation of constraints did not meaningfully improve fit, the more parsimonious model was retained. In the case of inadmissible variance estimates, such as negative variances, or clearly non‐informative parameter estimates, the respective parameter would be fixed to zero; however, the final selected models reported below yielded admissible solutions.

Based on the final selected model for each parameter, LST coefficients were computed. Reliability was defined as the proportion of observed variance attributable to all error‐free latent components. Common trait specificity was defined as the proportion of observed variance attributable to the stable trait component shared with the recovery reference condition. For resting indicators, total trait specificity was computed as common trait specificity plus method specificity. Occasion specificity was defined as the proportion of variance attributable to occasion‐specific state‐residuals. Method specificity was defined as the proportion of variance attributable to the resting‐specific method factor, that is, condition‐specific stable variance in seated rest relative to recovery. All SEM analyses were conducted in *R* 4.4.0 (R Core Team 2024) using the *lavaan* package (Rosseel 2012). The analysis code is available at https://osf.io/9fd5c/overview?view_only=f062892ada444ac6b4e3e0ccec7ab0d6.

## 3 Results

### 3.1 Descriptive Statistics

RMSSD, pNN50 and RR showed acceptable skewness and kurtosis and were used in the raw metric. Only HF power showed a non‐normal distribution (i.e., kurtosis > 7 and skewness > 2), therefore a log transformation was applied to it prior to the SEM analyses to reduce skewness and improve its distributional properties. Table 2 reports means and standard deviations of all parameters during recovery and resting at T1 and T2, with log‐transformed values additionally shown for HF power.

**TABLE 2:** Means (*M*) ± one standard deviation (SD) for heart rate variability parameters (raw and logarithmized).

Condition	Parameter	T1 (M1 ± SD1)		T2 (M2 ± SD2)	
		Raw	Logarithmized	Raw	Logarithmized
Resting	RMSSD	35.44 ± 22.12	—	36.97 ± 22.72	—
	pNN50	16.27 ± 16.71	—	16.69 ± 18.37	—
	HF power	539.15 ± 773.75	5.45 ± 1.51	522.31 ± 722.84	5.46 ± 1.37
	RR	859.26 ± 123.42	—	889.20 ± 118.85	—
Recovery	RMSSD	33.64 ± 19.05	—	34.62 ± 22.33	—
	pNN50	12.44 ± 13.56	—	13.45 ± 15.93	—
	HF power	398.67 ± 496.23	5.26 ± 1.44	468.02 ± 801.43	5.21 ± 1.60
	RR	856.50 ± 117.33	—	885.42 ± 115.97	—

*Note:* Logarithmized descriptive values are only shown for HF power, the only HRV parameter transformed for SEM analyses.

Abbreviations: HF power, high‐frequency power (ms^2^); pNN50, percent of difference between adjacent NN intervals that are greater than 50 ms (%); recovery, recumbent recovery interval directly after natural stressor (venipuncture); resting, seated resting condition approximately 20 min after the recovery period following the natural stressor (venipuncture); RMSSD, root mean square of successive differences (ms); RR, mean inter‐beat (RR) interval (ms).

### 3.2 Zero‐Order Test–Retest Associations

Zero‐order T1–T2 correlations (Table 3) indicated significant rank‐order stability across the one‐year interval for all cardiac and HRV‐derived parameters and both measurement conditions (all *p* < 0.001). For RMSSD, T1–T2 correlations were *r* = 0.60 during seated rest and *r* = 0.60 during recovery. For pNN50, the corresponding correlations were *r* = 0.61 and *r* = 0.65, respectively. HF power showed comparatively smaller associations, with *r* = 0.44 during rest and *r* = 0.48 during recovery. RR showed correlations of *r* = 0.61 during rest and *r* = 0.68 during recovery. Thus, RMSSD, pNN50, and RR showed moderate‐to‐strong preservation of individual rank ordering over 1 year, whereas HF power showed comparatively lower temporal rank‐order stability.

**TABLE 3:** Zero‐order correlations among cardiac and HRV‐derived parameters.

Variable	1	2	3	4	5	6	7	8	9	10	11	12	13	14	15
1. T1 Rest RMSSD															
2. T1 Recovery RMSSD	0.72***														
3. T2 Rest RMSSD	0.60***	0.57***													
4. T2 Recovery RMSSD	0.58***	0.60***	0.79***												
5. T1 Rest pNN50	0.95***	0.69***	0.59***	0.59***											
6. T1 Recovery pNN50	0.69***	0.93***	0.58***	0.62***	0.73***										
7. T2 Rest pNN50	0.57***	0.55***	0.95***	0.78***	0.61***	0.59***									
8. T2 Recovery pNN50	0.56***	0.60***	0.74***	0.94***	0.61***	0.65***	0.78***								
9. T1 Rest HF Power	0.80***	0.61***	0.49***	0.51***	0.84***	0.57***	0.48***	0.52***							
10. T1 Recovery HF Power	0.63***	0.82***	0.45***	0.50***	0.65***	0.74***	0.43***	0.50***	0.76***						
11. T2 Rest HF Power	0.47***	0.47***	0.87***	0.70***	0.49***	0.49***	0.83***	0.66***	0.44***	0.40***					
12. T2 Recovery HF Power	0.46***	0.50***	0.68***	0.82***	0.48***	0.52***	0.65***	0.76***	0.45***	0.48***	0.71***				
13. T1 Rest RR	0.47***	0.43***	0.22**	0.20**	0.42***	0.39***	0.24**	0.20*	0.28***	0.29***	0.09	0.14			
14. T1 Recovery RR	0.33***	0.47***	0.19*	0.20*	0.29***	0.44***	0.19*	0.20*	0.16*	0.32***	0.07	0.11	0.87***		
15. T2 Rest RR	0.31***	0.42***	0.60***	0.52***	0.30***	0.40***	0.59***	0.50***	0.21**	0.24**	0.45***	0.41***	0.61***	0.59***	
16. T2 Recovery RR	0.27***	0.39***	0.45***	0.55***	0.27***	0.37***	0.43***	0.53***	0.17*	0.22**	0.33***	0.40***	0.62***	0.68***	0.85***

Abbreviations: HF power, power in the high frequency band; pNN50, percent of difference between adjacent NN intervals that are greater than 50 ms (%); RMSSD, root mean square of successive differences; RR, mean inter‐beat (RR) interval (ms); T1, Time 1; T2, Time 2.

* *p* < 0.05;

** *p* < 0.01;

*** *p* < 0.001.

### 3.3 Conventional Test–Retest Reliability

Conventional test–retest ICCs are reported in Table 4. Consistency ICCs ranged from 0.44 to 0.68, whereas absolute‐agreement ICCs ranged from 0.44 to 0.66. For RMSSD, consistency and absolute‐agreement ICCs were identical or nearly identical, with estimates of 0.60 (95% CI [0.49, 0.69]) and 0.60 (95% CI [0.49, 0.68]) during rest and 0.59 (95% CI [0.48, 0.68]) for both definitions during recovery. For pNN50, both coefficients were 0.60 (95% CI [0.49, 0.69]) during rest and 0.64 (95% CI [0.54, 0.72]) during recovery. HF power showed lower test–retest reliability, with consistency and absolute‐agreement ICCs of 0.44 (95% CI [0.31, 0.55]) during rest and 0.47 (95% CI [0.35, 0.59]) during recovery. For RR, consistency ICCs were 0.61 (95% CI [0.51, 0.70]) during rest and 0.68 (95% CI [0.59, 0.76]) during recovery, whereas the corresponding absolute‐agreement ICCs were slightly lower, at 0.59 (95% CI [0.48, 0.69]) and 0.66 (95% CI [0.55, 0.75]), respectively. Overall, the close correspondence between consistency and absolute‐agreement coefficients indicates that incorporating systematic differences between measurement occasions had little influence on the estimated one‐year test–retest reliability.

**TABLE 4:** ICC estimates for consistency and absolute agreement across time 1 and time 2.

Measure	Condition	ICC type	Estimate	95% CI	*F*(df1, df2)
RMSSD	Rest	Consistency	0.60***	[0.49, 0.69]	*F*(168, 168) = 3.96***
	Rest	Agreement	0.60***	[0.49, 0.68]	*F*(168, 168.98) = 3.96***
	Recovery	Consistency	0.59***	[0.48, 0.68]	*F*(163, 163) = 3.90***
	Recovery	Agreement	0.59***	[0.48, 0.68]	*F*(163, 163.09) = 3.90***
pNN50	Rest	Consistency	0.60***	[0.49, 0.69]	*F*(154, 154) = 4.03***
	Rest	Agreement	0.60***	[0.49, 0.69]	*F*(154, 154.72) = 4.03***
	Recovery	Consistency	0.64***	[0.54, 0.72]	*F*(161, 161) = 4.54***
	Recovery	Agreement	0.64***	[0.54, 0.72]	*F*(161, 161.90) = 4.54***
HF power	Rest	Consistency	0.44***	[0.31, 0.55]	*F*(168, 168) = 2.57***
	Rest	Agreement	0.44***	[0.31, 0.55]	*F*(168, 168.04) = 2.57***
	Recovery	Consistency	0.47***	[0.35, 0.59]	*F*(161, 161) = 2.80***
	Recovery	Agreement	0.47***	[0.35, 0.59]	*F*(161, 161.44) = 2.80***
RR	Rest	Consistency	0.61***	[0.51, 0.70]	*F*(168, 168) = 4.14***
	Rest	Agreement	0.59***	[0.48, 0.69]	*F*(168, 119.02) = 4.14***
	Recovery	Consistency	0.68***	[0.59, 0.76]	*F*(161, 161) = 5.33***
	Recovery	Agreement	0.66***	[0.55, 0.75]	*F*(161, 93.29) = 5.33***

*Note:* Consistency and absolute‐agreement ICC estimates are reported. Noninteger degrees of freedom are rounded to two decimal places.

Abbreviations: CI, confidence interval; HF power, power in the high frequency band; ICC, intraclass correlation coefficient; pNN50, percent of difference between adjacent NN intervals that are greater than 50 ms (%); RMSSD, root mean square of successive differences; RR, mean inter‐beat (RR) interval (ms).

*** *p* < 0.001.

### 3.4 LST Models of HRV

The LST analyses had two main aims. First, we estimated the extent to which individual differences in each cardiac and HRV‐derived parameter reflected a stable transsituational trait, occasion‐specific deviations, condition‐specific method variance, and measurement error. Second, we quantified the reliability of each single measurement after separating measurement error from stable and occasion‐specific variance. Model‐fit statistics for all tested models are reported in Table 5. The final selected models were the restrictive M0 model for RMSSD, the fully relaxed M2 model for pNN50, the M1 model with freely estimated error variances for HF power, and the restrictive M0 model for RR.

**TABLE 5:** Fit indices for all LST models tested across vmHRV measures.

Measure	Model	*χ* ^2^	df	*p*	CFI	TLI	RMSEA	SRMR	AIC	BIC	Model comparison				
											Models	Δ*χ* ^2^	Δdf	*χ* ^2^ *p*	ΔCFI
RMSSD	**M0 (restrictive)**	**7.54**	**6**	**0.274**	**0.996**	**0.996**	**0.038**	**0.073**	**5747.38**	**5772.70**					
	M1 (free errors)	4.32	3	0.229	0.997	0.993	0.050	0.064	5750.16	5784.97	M1 vs. M0	3.22	3	0.358	0.001
	M2 (free errors + free states)	0.69	2	0.710	1.000	1.010	0.000	0.014	5748.53	5786.51	M2 vs. M1	3.63	1	0.057	0.003
pNN50	M0 (restrictive)	22.00	6	0.001	0.959	0.959	0.123	0.113	5230.51	5255.82					
	M1 (free errors)	9.12	3	0.028	0.984	0.968	0.108	0.087	5223.63	5258.44	M1 vs. M0	12.88	3	0.005	0.025
	**M2 (free errors + free states)**	**1.75**	**2**	**0.416**	**1.000**	**1.000**	**0.000**	**0.029**	**5218.25**	**5256.23**	**M2 vs. M1**	**7.37**	**1**	**0.007**	**0.016**
HF power	M0 (restrictive)	13.93	6	0.030	0.974	0.974	0.087	0.071	2170.20	2195.52					
	**M1 (free errors)**	**6.47**	**3**	**0.091**	**0.989**	**0.978**	**0.081**	**0.045**	**2168.75**	**2203.56**	**M1 vs. M0**	**7.46**	**3**	**0.059**	**0.015**
	M2 (free errors + free states)	6.12	2	0.047	0.987	0.960	0.109	0.043	2170.40	2208.38	M2 vs. M1	0.35	1	0.554	−0.002
RR	**M0 (restrictive)**	**7.50**	**6**	**0.277**	**0.997**	**0.997**	**0.038**	**0.028**	**7880.93**	**7906.25**					
	M1 (free errors)	3.42	3	0.331	0.999	0.998	0.028	0.040	7882.86	7917.67	M1 vs. M0	4.07	3	0.254	0.002
	M2 (free errors + free states)	3.14	2	0.208	0.998	0.994	0.057	0.034	7884.58	7922.55	M2 vs. M1	0.28	1	0.596	−0.001

*Note:* In bold the models selected as best fitting ones.

Abbreviations: Δ*χ* ^2^, difference in chi‐square test statistic among the compared models; ΔCFI, difference in CFI among the compared models; Δdf, difference in degrees of freedom among the compared models; *χ* ^2^, chi‐square test statistic; AIC, Akaike information criterion; BIC, Bayesian information criterion; CFI, comparative fit index; df, degrees of freedom; RMSEA, root mean square error of approximation; SRMR, standardized root mean square residual; TLI, Tucker–Lewis index.

For RMSSD, the restrictive M0 model, in which the four measurement‐error variances were constrained to equality and the two state‐residual variances were constrained to equality, showed good fit, *χ* ^2^(6) = 7.54, *p* = 0.274, CFI = 0.996, TLI = 0.996, RMSEA = 0.038, and SRMR = 0.073. Freeing the error variances in M1 did not significantly improve model fit, Δ*χ* ^2^(3) = 3.22, *p* = 0.358, ΔCFI = 0.001. Although M2 showed excellent descriptive fit after the state‐residual variances were additionally freed, its improvement over M1 did not reach statistical significance, Δ*χ* ^2^(1) = 3.63, *p* = 0.057, ΔCFI = 0.003. Moreover, M0 had the lowest AIC and BIC among the RMSSD models. Therefore, the more parsimonious M0 model was retained for RMSSD.

For pNN50, the restrictive M0 model showed inadequate fit, *χ* ^2^(6) = 22.00, *p* = 0.001, CFI = 0.959, TLI = 0.959, RMSEA = 0.123, and SRMR = 0.113. Freeing the error variances in M1 significantly improved model fit, Δ*χ* ^2^(3) = 12.88, *p* = 0.005, ΔCFI = 0.025. Nevertheless, the M1 model continued to show evidence of misfit, *χ* ^2^(3) = 9.12, *p* = 0.028, RMSEA = 0.108, and SRMR = 0.087. Freeing the two state‐residual variances in M2 produced a further significant improvement, Δ*χ* ^2^(1) = 7.37, *p* = 0.007, ΔCFI = 0.016.

The resulting M2 model showed excellent fit, *χ* ^2^(2) = 1.75, *p* = 0.416, CFI = 1.000, TLI = 1.000, RMSEA = 0.000, and SRMR = 0.029, and had the lowest AIC among the pNN50 models. Accordingly, M2 was retained as the final model for pNN50.

For HF power, the restrictive M0 model showed some evidence of inadequate fit, *χ* ^2^(6) = 13.93, *p* = 0.030, CFI = 0.974, TLI = 0.974, RMSEA = 0.087, and SRMR = 0.071. Freeing the measurement‐error variances improved fit, Δ*χ* ^2^(3) = 7.46, *p* = 0.059, ΔCFI = 0.015, and yielded an M1 model with a nonsignificant chi‐square test, strong incremental‐fit indices, and a low residual‐based fit index, *χ* ^2^(3) = 6.47, *p* = 0.091, CFI = 0.989, TLI = 0.978, RMSEA = 0.081, and SRMR = 0.045.

Although RMSEA was marginally above the prespecified 0.08 criterion, the remaining fit indices and the lower AIC supported M1 over M0. Freeing the state‐residual variances in M2 did not further improve fit, Δ*χ* ^2^(1) = 0.35, *p* = 0.554, ΔCFI = −0.002, and resulted in less favorable RMSEA and information criteria. Therefore, M1 was retained for HF power.

For RR, the restrictive M0 model showed good fit, *χ* ^2^(6) = 7.50, *p* = 0.277, CFI = 0.997, TLI = 0.997, RMSEA = 0.038, and SRMR = 0.028. Freeing the error variances did not significantly improve fit, Δ*χ* ^2^(3) = 4.07, *p* = 0.254, ΔCFI = 0.002. Similarly, freeing the state‐residual variances did not improve M2 relative to M1, Δ*χ* ^2^(1) = 0.28, *p* = 0.596, ΔCFI = −0.001. M0 also had the lowest AIC and BIC among the RR models. The restrictive M0 model was therefore retained for RR.

Unstandardized parameter estimates from the final selected models are presented in Table 6. Positive trait‐variance estimates were obtained for all four parameters, indicating the presence of stable between‐person differences across the one‐year interval and across the recovery and resting conditions. The final RMSSD and RR models supported equality of both state‐residual and measurement‐error variances across the design. In contrast, the final pNN50 model required both state‐residual and error variances to differ across occasions and conditions, indicating a less homogeneous variance structure. For HF power, the state‐residual variances could be constrained to equality, whereas measurement‐error variances differed across the four indicators. Method variance was estimated for the non‐reference resting condition for all parameters, although it represented a relatively small component of total variance.

**TABLE 6:** Estimated model parameters of the latent state–trait model of the cardiac and HRV‐derived parameters for the two measurement occasions (T1 vs. T2) and the two measurement conditions (resting vs. recovery).

	RMSSD		pNN50		HF power (ln)		RR	
	Estimate (SE)	95% CI	Estimate (SE)	95% CI	Estimate (SE)	95% CI	Estimate (SE)	95% CI
Trait	267.68*** (45.25)	[180.30, 358.64]	147.17*** (23.70)	[100.60, 195.57]	0.91*** (0.15)	[0.59, 1.18]	8362.95*** (1279.23)	[5889.86, 10937.30]
State‐residual 1	92.28*** (23.27)	[50.16, 141.05]	11.96 (14.42)	[−14.95, 43.05]	0.67 (0.34)	[0.13, 1.43]	3718.83*** (589.37)	[2625.50, 4943.16]
State‐residual 2	92.28*** (23.27)	[50.16, 141.05]	80.81*** (20.39)	[41.08, 120.64]	0.67 (0.34)	[0.13, 1.43]	3718.83*** (589.37)	[2625.50, 4943.16]
Method	30.32 (20.88)	[−9.92, 71.34]	22.19* (9.83)	[4.14, 42.04]	0.07 (0.07)	[−0.05, 0.22]	1160.23*** (335.39)	[531.04, 1843.68]
Error 1	94.57*** (16.68)	[63.31, 128.19]	74.93** (24.74)	[29.97, 128.44]	0.54*** (0.16)	[0.22, 0.87]	1415.71*** (152.10)	[1122.35, 1713.47]
Error 2	94.57*** (16.68)	[63.31, 128.19]	31.18** (9.85)	[12.78, 51.02]	0.43*** (0.10)	[0.23, 0.63]	1415.71*** (152.10)	[1122.35, 1713.47]
Error 3	94.57*** (16.68)	[63.31, 128.19]	78.65*** (17.03)	[44.47, 111.19]	0.39** (0.12)	[0.14, 0.64]	1415.71*** (152.10)	[1122.35, 1713.47]
Error 4	94.57*** (16.68)	[63.31, 128.19]	30.45* (12.66)	[6.18, 57.04]	0.85* (0.42)	[0.20, 1.81]	1415.71*** (152.10)	[1122.35, 1713.47]

*Note:* All reported statistics are based on maximum likelihood estimates. Cells indicate estimates with standard errors in parentheses. 95% Bootstrapped confidence intervals are presented in squared brackets.

Abbreviations: error_*i*_, measurement errors of the measured variables; HF power, power in the high frequency band (ms^2^); method, condition‐specific variance related to the non‐reference condition (resting), modeled relative to recovery; pNN50, percent of difference between adjacent NN intervals that are greater than 50 ms (%); RMSSD, root mean square of successive differences (ms); RR, mean inter‐beat (RR) interval (ms); state‐residual_i_, effects of the respective measurement occasion and person‐occasion interaction on the respective latent state variable; Trait, individual differences that are consistent across the two measurement time points and the two conditions.

* *p* < 0.05;

** *p* < 0.01;

*** *p* < 0.001.

Across all parameters, conditions, and occasions, reliability ranged from 0.650 to 0.903, as depicted in Table 7. RR showed the highest and most consistent reliability, with estimates of 0.895 during recovery and 0.903 during rest at both measurement occasions.

**TABLE 7:** Reliability, expected trait, occasion, and method specificity of the cardiac and HRV‐derived parameters.

Measure	Condition	Occasion	TraSpec common	TraSpec total	OccSpec	MethSpec	Rel
RMSSD	Recovery	T1	0.589 [0.453, 0.691]	—	0.203 [0.110, 0.308]	—	0.792 [0.728, 0.848]
		T2	0.589 [0.453, 0.691]	—	0.203 [0.110, 0.308]	—	0.792 [0.728, 0.848]
	Rest	T1	0.552 [0.415, 0.670]	0.615 [0.496, 0.718]	0.190 [0.105, 0.286]	0.063 [−0.022, 0.139]	0.805 [0.738, 0.864]
		T2	0.552 [0.415, 0.670]	0.615 [0.496, 0.718]	0.190 [0.105, 0.286]	0.063 [−0.022, 0.139]	0.805 [0.738, 0.864]
pNN50	Recovery	T1	0.773 [0.606, 0.932]	—	0.063 [−0.089, 0.214]	—	0.836 [0.737, 0.930]
		T2	0.569 [0.453, 0.690]	—	0.313 [0.174, 0.443]	—	0.882 [0.788, 0.973]
	Rest	T1	0.574 [0.428, 0.743]	0.661 [0.504, 0.817]	0.047 [−0.066, 0.163]	0.087 [0.014, 0.177]	0.708 [0.562, 0.867]
		T2	0.448 [0.335, 0.558]	0.515 [0.411, 0.630]	0.246 [0.137, 0.342]	0.067 [0.012, 0.135]	0.761 [0.669, 0.854]
HF power	Recovery	T1	0.455 [0.253, 0.678]	—	0.333 [0.084, 0.588]	—	0.787 [0.675, 0.880]
		T2	0.375 [0.194, 0.588]	—	0.275 [0.058, 0.537]	—	0.650 [0.441, 0.901]
	Rest	T1	0.416 [0.228, 0.638]	0.451 [0.247, 0.698]	0.305 [0.077, 0.534]	0.034 [−0.025, 0.107]	0.755 [0.636, 0.874]
		T2	0.447 [0.256, 0.664]	0.484 [0.265, 0.735]	0.327 [0.079, 0.578]	0.037 [−0.026, 0.123]	0.811 [0.699, 0.922]
RR	Recovery	T1	0.620 [0.510, 0.707]	—	0.276 [0.202, 0.370]	—	0.895 [0.858, 0.922]
		T2	0.620 [0.510, 0.707]	—	0.276 [0.202, 0.370]	—	0.895 [0.858, 0.922]
	Rest	T1	0.571 [0.463, 0.653]	0.650 [0.553, 0.731]	0.254 [0.182, 0.337]	0.079 [0.037, 0.123]	0.903 [0.870, 0.929]
		T2	0.571 [0.463, 0.653]	0.650 [0.553, 0.731]	0.254 [0.182, 0.337]	0.079 [0.037, 0.123]	0.903 [0.870, 0.929]

*Note:* All reported statistics are based on maximum likelihood estimates. 95% Bootstrapped confidence intervals are presented in squared brackets.

Abbreviations: HF power, power in the high frequency band; MethSpec, method specificity of the parameter; OccSpec, occasion specificity of the parameter; pNN50, percent of difference between adjacent NN intervals that are greater than 50 ms (%); Rel, reliability of the parameter; RMSSD, root mean square of successive differences; RR, mean inter‐beat (RR) interval (ms); TraSpec Total, total trait specificity of the Rest parameters, considering the method specificity; TraSpec, trait specificity of the parameter (here, common refers to the shared variance that the Rest condition has in common with the Recovery loadings, without considering the method specificity).

RMSSD also showed consistently high reliability, with values of 0.792 during recovery and 0.805 during rest. Reliability for pNN50 ranged from 0.708 to 0.882, whereas HF power showed the broadest and generally lowest range, from 0.650 during recovery at T2 to 0.811 during rest at T2. Trait specificity, represented by common trait specificity for recovery and total trait specificity for rest, ranged from 0.375 to 0.773. RR showed the most consistently trait‐like profile across occasions and conditions. Trait specificity was 0.620 during recovery, and total trait specificity was 0.650 during rest, with identical values at T1 and T2. RMSSD showed a similarly consistent pattern: trait specificity was 0.589 during recovery and total trait specificity was 0.615 during rest at both occasions. pNN50 showed the highest single trait‐specificity estimate but also the clearest heterogeneity across measurement occasions.

During recovery, trait specificity decreased from 0.773 at T1 to 0.569 at T2. During rest, total trait specificity decreased from 0.661 at T1 to 0.515 at T2. Thus, pNN50 was strongly trait‐like at T1, whereas a larger proportion of its reliable variance was occasion‐specific at T2. HF power showed the lowest trait‐specificity estimates overall. Trait specificity ranged from 0.375 to 0.455 during recovery, while total trait specificity ranged from 0.451 to 0.484 during rest. Occasion specificity ranged from 0.047 to 0.333. RMSSD showed relatively modest and highly consistent occasion specificity, with values of 0.203 during recovery and 0.190 during rest at both measurement occasions. RR showed moderate occasion specificity, with values of 0.276 during recovery and 0.254 during rest. For pNN50, occasion specificity differed markedly between occasions. It was low at T1, with values of 0.063 during recovery and 0.047 during rest, but increased at T2 to 0.313 during recovery and 0.246 during rest. HF power showed the highest and most consistently elevated occasion‐specific components, ranging from 0.275 to 0.333 across conditions and occasions.

Method specificity was estimated only for the non‐reference resting condition and was small across all parameters, ranging from 0.034 to 0.087. HF power showed the smallest method‐specific component, with estimates of 0.034 at T1 and 0.037 at T2. Method specificity was 0.063 for RMSSD and 0.079 for RR at both occasions. pNN50 showed the largest method‐specific estimate at T1 (0.087), followed by 0.067 at T2. For every parameter, method specificity was substantially smaller than the corresponding trait‐specific component, indicating that stable resting‐specific deviations from the recovery reference condition explained a relatively limited proportion of observed variance.

Overall, the LST models indicated that all four cardiac and HRV‐derived parameters contained substantial reliable and trait‐like variance across the one‐year interval.

RR and RMSSD showed the most consistent psychometric profiles across occasions and conditions. pNN50 showed the highest individual trait‐specificity estimate but also the greatest temporal heterogeneity, with lower trait specificity and greater occasion specificity at T2. HF power contained meaningful stable variance but was comparatively more sensitive to occasion‐specific and measurement‐related influences. Condition‐specific method variance was consistently small, suggesting that individual differences generalized substantially across recumbent recovery and seated rest.

## 4 Discussion

Although vmHRV is widely used in research and clinical contexts as an indicator of physical and psychological health, its psychometric robustness remains insufficiently established, despite being a prerequisite for its interpretation as a biomarker. The present study addressed this issue by examining the temporal and transsituational stability of three established vmHRV indices, RMSSD, pNN50, and HF power, and mean RR interval over a one‐year period in a population‐based adult sample. By applying STMSM(M–1) models, we decomposed observed variance into stable trait‐like individual differences, occasion‐specific fluctuations, condition‐specific method variance, and measurement error.

Overall, all four parameters contained substantial reliable variance, with reliability estimates ranging from 0.650 to 0.903. Trait specificity ranged from 0.375 to 0.773, indicating that a meaningful proportion of individual differences was stable across the one‐year interval and generalized across recovery and resting conditions. At the same time, occasion specificity ranged from 0.047 to 0.333, demonstrating that the measures were not pure trait indicators. Instead, observed scores reflected parameter‐specific combinations of stable individual differences and occasion‐dependent physiological influences. Method specificity was comparatively small, ranging from 0.034 to 0.087, suggesting that systematic resting‐specific deviations from the recovery reference condition were present but did not dominate the variance structure.

RR showed the most consistently trait‐like psychometric profile across the complete design. Its reliability was 0.895 during recovery and 0.903 during rest, whereas trait specificity was 0.620 during recovery and total trait specificity was 0.650 during rest. These estimates were identical across the two measurement occasions. Thus, individual differences in mean cardiac timing were highly reproducible across a one‐year interval and across the two assessment conditions. This finding also highlights an important conceptual distinction: high temporal and transsituational stability is not itself evidence of vagal specificity. RR reflects mean cardiac timing and broader autonomic regulation, whereas RMSSD, pNN50, and HF power are more specifically interpreted as indices of cardiac vagal modulation. Consequently, the psychometric quality of a measure and the physiological specificity of its interpretation should be considered separately.

Among the established vmHRV indices, RMSSD showed the most consistent overall psychometric profile. Reliability was 0.792 during recovery and 0.805 during rest; trait specificity was 0.589 during recovery, and total trait specificity was 0.615 during rest.

Occasion specificity was comparatively modest and highly consistent, ranging from 0.190 to 0.203, while method specificity was 0.063. The restrictive model, in which both state‐residual and error variances were equal across the design, adequately represented the RMSSD data. Together, these findings indicate that the composition of RMSSD variance remained highly similar across measurement occasions and conditions and that most of its reliable variance reflected stable individual differences. RMSSD therefore appears to provide a comparatively robust indicator of individual differences in cardiac vagal regulation over a one‐year period.

pNN50 showed a different and more heterogeneous pattern. It produced the highest individual trait‐specificity estimate in the study, with 0.773 of the recovery variance attributable to stable trait differences at T1. Total trait specificity during rest at T1 was also high at 0.661. However, these values declined at T2 to 0.569 during recovery and 0.515 during rest. Correspondingly, occasion specificity increased from 0.063 to 0.313 during recovery and from 0.047 to 0.246 during rest. Reliability nevertheless remained moderate to high, ranging from 0.708 to 0.882, because both trait and occasion‐specific variance constitute reliable variance within the LST framework.

The need to freely estimate both measurement‐error and state‐residual variances in the final pNN50 model further indicates that its variance composition was less homogeneous across the design than that of RMSSD or RR. At T1, individual differences in pNN50 were predominantly trait‐like, whereas at T2 a considerably larger proportion reflected occasion‐specific influences. Therefore, the high trait specificity observed at one occasion should not be interpreted as evidence of uniform long‐term stability across all measurements. Although pNN50 reliably differentiated individuals, the relative contributions of stable and occasion‐specific variance changed over time. This temporal heterogeneity may be relevant when pNN50 is used for individual‐level classification or longitudinal monitoring. A single observation may primarily reflect stable individual differences under some circumstances but contain a substantially larger occasion‐specific component under others. Repeated assessments may consequently be particularly valuable for pNN50, allowing enduring individual differences to be distinguished from temporary physiological deviations.

HF power showed the weakest trait‐like profile among the vmHRV indices. Trait specificity ranged from 0.375 to 0.455 during recovery, and total trait specificity ranged from 0.451 to 0.484 during rest. Occasion specificity was relatively high and consistent, ranging from 0.275 to 0.333, whereas reliability ranged from 0.650 to 0.811.

These findings indicate that HF power contained meaningful stable individual‐difference variance but was more sensitive than RMSSD to occasion‐specific and measurement‐related influences. Consistent with this interpretation, the final HF power model required measurement‐error variances to differ across indicators, although the state‐residual variances could be constrained to equality. The HF power findings are compatible with the methodological and physiological sensitivity of frequency‐domain HRV indices to respiratory behavior, short‐term stationarity, and spectral estimation procedures. In particular, respiratory frequency and depth can influence the amount of variability represented within the high‐frequency band. Because respiration was not directly measured in the present study, unmeasured respiratory differences may have contributed both to occasion‐specific variance and to differences in measurement error. Although HF power remained reliable and partly trait‐like, its interpretation as a stable individual‐difference marker may therefore require more stringent physiological standardization than RMSSD.

Condition‐specific method effects were small for all parameters. Because recovery was selected as the reference condition, method specificity represents stable variance in seated rest that was not shared with the recovery‐reference trait. HF power showed the smallest method‐specific component, followed by RMSSD, RR, and pNN50. Even the largest estimate, 0.087 for pNN50 at T1, was modest relative to the corresponding trait component. This pattern indicates substantial transsituational generalizability across recumbent post‐venipuncture recovery and seated rest. Nevertheless, the presence of nonzero method specificity confirms that posture and contextual features of the assessment contributed systematically, albeit modestly, to observed individual differences.

The interpretation of method specificity is necessarily relative to the selected reference condition. In an M–1 model, the trait factor is defined by the reference method and therefore contains the stable variance associated with recovery. The explicit method factor captures only the additional stable component of seated rest that is not shared with recovery. Consequently, the small method‐specificity estimates should not be interpreted as evidence that recovery and rest are physiologically equivalent or that recovery is free of condition‐specific influences. Rather, they indicate that comparatively little additional stable variance was unique to seated rest after accounting for the recovery‐reference trait.

In addition, the supplementary conventional test–retest analyses further clarify the meaning of temporal stability in the present study. Zero‐order T1–T2 correlations showed moderate‐to‐strong preservation of individual rank ordering for RMSSD, pNN50, and RR, with comparatively lower associations for HF power. Consistency and absolute‐agreement ICCs were highly similar across parameters and conditions, indicating that the estimated one‐year reproducibility was little affected by whether systematic differences between T1 and T2 were incorporated into the reliability coefficient. RMSSD, pNN50, and RR showed the strongest conventional test–retest coefficients, whereas HF power showed comparatively lower values. These ICCs nevertheless address a different question from the LST decomposition. Conventional ICCs summarize the reproducibility of individual differences across the two observed measurements, whereas the LST model separates stable trait‐related variance from occasion‐specific, method‐specific, and measurement‐error components. Accordingly, the two approaches provide complementary rather than interchangeable information about the temporal properties of HRV.

By the criteria of Koo and Li (2016), one‐year test–retest reliability was moderate for RMSSD, pNN50, and RR, and poor for HF power. The 95% confidence intervals of RMSSD, pNN50, and RR overlapped substantially, whereas HF power showed consistently lower point estimates, with intervals overlapping only marginally with those of RR during recovery. The LST results converge with this pattern: trait specificity exceeded 0.50 for RMSSD, pNN50, and RR, but remained below 0.50 for HF power, where occasion‐specific variance was comparatively large.

To summarize, and consistent with Bertsch et al. (2012), who examined RMSSD, pNN50, and HF power over a considerably shorter interval, vmHRV reflected both stable individual differences and occasion‐specific variation. The present study extends their work by demonstrating trait‐like variance across a one‐year interval, using a population‐based adult sample, and explicitly modeling condition‐specific variance through an STMSM(M–1) specification. The current findings further reveal distinct psychometric profiles among the vmHRV indices: RMSSD showed the most consistent balance of trait, occasion, and error variance; pNN50 reached the highest trait‐specificity estimate but varied more markedly across occasions; and HF power was comparatively more occasion‐sensitive.

The results have important implications for the interpretation of vmHRV as a biomarker. A useful biomarker should provide sufficiently reliable between‐person differentiation while retaining the capacity to reflect meaningful physiological variation. RMSSD appears especially promising in this regard. It showed robust reliability, substantial and consistent trait specificity, limited method specificity, and a modest but nonzero occasion‐specific component. This combination suggests that RMSSD reflects a relatively stable individual characteristic while retaining sensitivity to temporary physiological variation, supporting its use in clinical and health‐related research (Jarczok et al. 2022; Watanabe et al. 2002). pNN50 also showed substantial biomarker potential, but its greater temporal heterogeneity suggests that its interpretation may depend more strongly on the measurement occasion. HF power, although reliable and partly trait‐like, may require particularly careful standardization of respiratory and recording conditions. More generally, the present findings demonstrate that conclusions concerning the “stability of HRV” should not be generalized across parameters without considering their distinct variance structures.

The observed occasion‐specific variance should not be treated merely as unwanted noise. Within LST theory, occasion specificity reflects systematic state‐residuals rather than random measurement error. It may therefore represent meaningful temporary physiological states, contextual demands, person‐by‐occasion interactions, or other time‐varying influences. This distinction is central to vmHRV research because cardiac vagal activity is theoretically expected to function both as a relatively stable individual‐difference characteristic and as a dynamically responsive physiological process. The present findings empirically support this dual interpretation. This duality is also relevant to the increasing use of wearable devices and ambulatory HRV monitoring. Wearable‐derived stress, readiness, and recovery scores frequently rely on short‐term RMSSD. The current results indicate that RMSSD is strongly anchored in stable between‐person differences. A single RMSSD value therefore largely reflects a person's characteristic level rather than their current state, which limits its interpretation without reference to the individual baseline.

At the same time, its occasion‐specific component supports the use of repeated within‐person measurements to identify deviations from an individual baseline. Indeed, daily‐life research indicates that within‐person variation in RMSSD is associated with momentary states such as exhaustion (Wekenborg et al. 2025). HRV‐based monitoring may therefore be most informative when repeated observations are evaluated relative to a person's own longitudinal distribution.

Several limitations should be acknowledged. First, although the one‐year interval substantially extends previous LST investigations of vmHRV, the design included only two measurement occasions. Accordingly, the latent trait component should be understood as representing individual differences that were stable across the observed one‐year interval, rather than an invariant physiological characteristic. Additional waves would permit the examination of autoregressive processes, nonlinear stability patterns, and possible changes in the latent trait itself. They would also provide a stronger basis for determining whether the increased occasion specificity observed for pNN50 at T2 reflects a replicable temporal pattern or sample‐specific variation.

Second, the two conditions differed simultaneously in posture, temporal position within the laboratory protocol, and physiological context. Recovery was assessed in a recumbent position immediately following venipuncture, whereas rest was assessed while seated approximately 20 min later. The method factor captured the combined systematic influence of these differences but could not distinguish the specific effects of posture, post‐stressor recovery, elapsed time, or psychological context. Experimental studies varying these characteristics independently would be required to identify their separate contributions.

Third, respiration was not measured or modeled directly. This limitation is particularly relevant to HF power because individual differences and occasion‐specific changes in respiratory frequency and depth may affect high‐frequency spectral estimates. Future investigations should incorporate direct respiratory assessment or paced‐breathing conditions when the aim is to isolate cardiac vagal contributions to frequency‐domain HRV.

Fourth, because recovery was designated as the reference condition, the method‐effect estimates are reference‐dependent. Reversing the reference condition would change the interpretation of the trait and method‐specific components, although it would not change the observed covariance structure. The present estimates should therefore be interpreted specifically as quantifying resting‐specific deviations relative to recovery, rather than as representing an absolute or symmetric effect of measurement condition.

Finally, although the sample was population‐based and more heterogeneous than samples used in many previous reliability studies, participants were adults from a specific regional cohort who contributed sufficient physiological data to the longitudinal analyses. Moreover, health characteristics such as cardiovascular disease or cardioactive medication have been shown to affect the reliability of short‐term HRV measurements (Sandercock et al. 2005). Whether the variance components estimated here differ as a function of such characteristics could not be tested within the present design and warrants dedicated investigation, for example using multigroup or moderated LST models. Replication in clinical populations, older adults, younger age groups, and more ethnically and socioeconomically diverse samples is necessary before the estimated variance components can be generalized broadly.

In conclusion, the present findings support the interpretation of vmHRV as both trait‐like and state‐sensitive. RMSSD showed the most consistent psychometric profile among the vagally mediated indices, combining substantial trait specificity with high reliability and relatively modest occasion‐ and condition‐specific variance. pNN50 showed very high trait specificity under some conditions but greater heterogeneity across occasions, whereas HF power was more strongly influenced by occasion‐specific factors. RR showed the highest and most consistent stability overall, although this stability should not be equated with vagal specificity. Condition‐specific method variance was small for all parameters, indicating substantial generalization across recovery and seated rest. These findings reinforce the value of LST modeling for distinguishing stable individual differences from meaningful occasion‐related fluctuations and measurement error, and they underscore the importance of standardized protocols and repeated assessments when vmHRV is used as a physiological biomarker.

## Author Contributions

**Magdalena K. Wekenborg:** conceptualization, methodology, project administration, supervision, data curation, investigation, writing – original draft. **Clemens Kirschbaum:** conceptualization, writing – review and editing, supervision, resources. **LaBarron K. Hill:** conceptualization, methodology, writing – review and editing. **R. Arne Wittling:** conceptualization, methodology, formal analysis, writing – review and editing. **Julian Eder:** conceptualization, investigation, methodology, writing – review and editing, data curation. **Lorenzo Filosa:** methodology, formal analysis, writing – review and editing, supervision. **Julian F. Thayer:** conceptualization, methodology, writing – review and editing, supervision.

## Funding

This work was supported by the TU Dresden's Institutional Strategy (“The Synergetic University”), which is funded by the Excellence Initiative of the German Federal and State Governments.

## Ethics Statement

The study was conducted in accordance with the Declaration of Helsinki. The study protocol was approved by the ethics committee of TUD Dresden University of Technology (EK236062014).

## Consent

Written informed consent was obtained from all participants.

## Conflicts of Interest

R. Arne Wittling is managing director and majority shareholder of the Center for Neuroscience Research NPO, Trier (Forschungszentrum für innovative Neurotechnologien GmbH), which develops methods for heart rate variability and autonomic nervous system analysis, among other areas. The Center for Neuroscience Research NPO contributed to HRV preprocessing and parameter extraction using the NEUROCOR precision HRV algorithm. The other authors declare no conflicts of interest.

## Data Availability

The data analyzed in this study were collected within the Dresden Burnout Study and are subject to data protection regulations governing this cohort. Because the data contain potentially identifying health‐related information, they cannot be made publicly available. De‐identified data are available from the corresponding author upon reasonable request, subject to a data use agreement. Analysis code is available at https://osf.io/9fd5c/overview?view_only=f062892ada444ac6b4e3e0ccec7ab0d6.

## References

[psyp70445-bib-0001] Appelhans, B. M. , and L. J. Luecken . 2006. “Heart Rate Variability as an Index of Regulated Emotional Responding.” Review of General Psychology 10, no. 3: 229–240. 10.1037/1089-2680.10.3.229.

[psyp70445-bib-0002] Azulay, N. , R. B. Olsen , C. S. Nielsen , et al. 2022. “Reduced Heart Rate Variability Is Related to the Number of Metabolic Syndrome Components and Manifest Diabetes in the Sixth Tromsø Study 2007–2008.” Scientific Reports 12, no. 1: 11998. 10.1038/s41598-022-15824-0. PMC9283528 35835836

[psyp70445-bib-0003] Bertsch, K. , D. Hagemann , E. Naumann , H. Schächinger , and A. Schulz . 2012. “Stability of Heart Rate Variability Indices Reflecting Parasympathetic Activity.” Psychophysiology 49, no. 5: 672–682. 10.1111/j.1469-8986.2011.01341.x. 22335779

[psyp70445-bib-0004] Besson, C. , A. L. Baggish , P. Monteventi , L. Schmitt , F. Stucky , and V. Gremeaux . 2025. “Assessing the Clinical Reliability of Short‐Term Heart Rate Variability: Insights From Controlled Dual‐Environment and Dual‐Position Measurements.” Scientific Reports 15, no. 1: 5611. 10.1038/s41598-025-89892-3. PMC11829968 39955401

[psyp70445-bib-0005] Borges, C. , K. J. Mathewson , and L. A. Schmidt . 2018. “Short‐Term Test‐Retest Reliability of Respiratory Sinus Arrhythmia (RSA) in Young Adults.” Journal of Psychophysiology 32, no. 3: 85–96. 10.1027/0269-8803/a000187.

[psyp70445-bib-0006] Burleson, M. H. , K. M. Poehlmann , L. C. Hawkley , et al. 2003. “Neuroendocrine and Cardiovascular Reactivity to Stress in Mid‐Aged and Older Women: Long‐Term Temporal Consistency of Individual Differences.” Psychophysiology 40, no. 3: 358–369. 10.1111/1469-8986.00039. 12946110

[psyp70445-bib-0007] Cheng, Y.‐C. , M.‐I. Su , C.‐W. Liu , Y.‐C. Huang , and W.‐L. Huang . 2022. “Heart Rate Variability in Patients With Anxiety Disorders: A Systematic Review and Meta‐Analysis.” Psychiatry and Clinical Neurosciences 76, no. 7: 292–302. 10.1111/pcn.13356. 35340102

[psyp70445-bib-0008] Cheung, G. W. , and R. B. Rensvold . 2002. “Evaluating Goodness‐Of‐Fit Indexes for Testing Measurement Invariance.” Structural Equation Modeling: A Multidisciplinary Journal 9, no. 2: 233–255. 10.1207/S15328007SEM0902_5.

[psyp70445-bib-0009] De Vet, H. C. , C. B. Terwee , D. L. Knol , and L. M. Bouter . 2006. “When to Use Agreement Versus Reliability Measures.” Journal of Clinical Epidemiology 59, no. 10: 1033–1039. 10.1016/j.jclinepi.2005.10.015. 16980142

[psyp70445-bib-0010] Dragomir, A. I. , C. Gentile , R. P. Nolan , and B. D'Antono . 2014. “Three‐Year Stability of Cardiovascular and Autonomic Nervous System Responses to Psychological Stress.” Psychophysiology 51, no. 9: 921–931. 10.1111/psyp.12231. 24853995

[psyp70445-bib-0011] Fang, S.‐C. , Y.‐L. Wu , and P.‐S. Tsai . 2020. “Heart Rate Variability and Risk of All‐Cause Death and Cardiovascular Events in Patients With Cardiovascular Disease: A Meta‐Analysis of Cohort Studies.” Biological Research for Nursing 22, no. 1: 45–56. 10.1177/1099800419877442. 31558032

[psyp70445-bib-0012] Geiser, C. , and G. Lockhart . 2012. “A Comparison of Four Approaches to Account for Method Effects in Latent State–Trait Analyses.” Psychological Methods 17, no. 2: 255–283. 10.1037/a0026977. PMC3368086 22309958

[psyp70445-bib-0013] Gerritsen, J. , J. M. Dekker , B. J. TenVoorde , et al. 2001. “Impaired Autonomic Function Is Associated With Increased Mortality, Especially in Subjects With Diabetes, Hypertension, or a History of Cardiovascular Disease: The Hoorn Study.” Diabetes Care 24, no. 10: 1793–1798. 10.2337/diacare.24.10.1793. 11574444

[psyp70445-bib-0014] Gloger, E. M. , G. T. Smith , and S. C. Segerstrom . 2020. “Stress Physiology and Physiometrics.” In Handbook of Research Methods in Health Psychology. Routledge. 10.4324/9780429488320-13.

[psyp70445-bib-0015] Goedhart, A. D. , S. Van Der Sluis , J. H. Houtveen , G. Willemsen , and E. J. C. De Geus . 2007. “Comparison of Time and Frequency Domain Measures of RSA in Ambulatory Recordings.” Psychophysiology 44, no. 2: 203–215. 10.1111/j.1469-8986.2006.00490.x. 17343704

[psyp70445-bib-0016] Grol, M. , and R. De Raedt . 2020. “The Link Between Resting Heart Rate Variability and Affective Flexibility.” Cognitive, Affective, & Behavioral Neuroscience 20, no. 4: 746–756. 10.3758/s13415-020-00800-w. 32462431

[psyp70445-bib-0017] Guijt, A. M. , J. K. Sluiter , and M. H. W. Frings‐Dresen . 2007. “Test‐Retest Reliability of Heart Rate Variability and Respiration Rate at Rest and During Light Physical Activity in Normal Subjects.” Archives of Medical Research 38, no. 1: 113–120. 10.1016/j.arcmed.2006.07.009. 17174734

[psyp70445-bib-0018] Hagemann, D. , J. Hewig , J. Seifert , E. Naumann , and D. Bartussek . 2005. “The Latent State‐Trait Structure of Resting EEG Asymmetry: Replication and Extension.” Psychophysiology 42, no. 6: 740–752. 10.1111/j.1469-8986.2005.00367.x. 16364070

[psyp70445-bib-0019] Hagemann, D. , E. Naumann , J. F. Thayer , and D. Bartussek . 2002. “Does Resting Electroencephalograph Asymmetry Reflect a Trait? An Application of Latent State‐Trait Theory.” Journal of Personality and Social Psychology 82, no. 4: 619–641. 10.1037/0022-3514.82.4.619. 11999928

[psyp70445-bib-0020] Hellhammer, J. , E. Fries , O. W. Schweisthal , W. Schlotz , A. A. Stone , and D. Hagemann . 2007. “Several Daily Measurements Are Necessary to Reliably Assess the Cortisol Rise After Awakening: State‐ and Trait Components.” Psychoneuroendocrinology 32, no. 1: 80–86. 10.1016/j.psyneuen.2006.10.005. 17127010

[psyp70445-bib-0021] Hermes, M. , D. Hagemann , P. Britz , et al. 2009. “Latent State–Trait Structure of Cerebral Blood Flow in a Resting State.” Biological Psychology 80, no. 2: 196–202. 10.1016/j.biopsycho.2008.09.003. 18838099

[psyp70445-bib-0022] Hildebrandt, L. K. , C. McCall , H. G. Engen , and T. Singer . 2016. “Cognitive Flexibility, Heart Rate Variability, and Resilience Predict Fine‐Grained Regulation of Arousal During Prolonged Threat.” Psychophysiology 53, no. 6: 880–890. 10.1111/psyp.12632. 26899260

[psyp70445-bib-0023] Hu, M. X. , F. Lamers , B. W. J. H. Penninx , and E. J. C. de Geus . 2017. “Temporal Stability and Drivers of Change in Cardiac Autonomic Nervous System Activity.” Autonomic Neuroscience 208: 117–125. 10.1016/j.autneu.2017.07.005. 28774803

[psyp70445-bib-0024] Jandackova, V. K. , A. Britton , M. Malik , and A. Steptoe . 2016. “Heart Rate Variability and Depressive Symptoms: A Cross‐Lagged Analysis Over a 10‐Year Period in the Whitehall II Study.” Psychological Medicine 46, no. 10: 2121–2131. 10.1017/S003329171600060X. 27181276

[psyp70445-bib-0025] Jarczok, M. N. , K. Weimer , C. Braun , et al. 2022. “Heart Rate Variability in the Prediction of Mortality: A Systematic Review and Meta‐Analysis of Healthy and Patient Populations.” Neuroscience & Biobehavioral Reviews 143: 104907. 10.1016/j.neubiorev.2022.104907. 36243195

[psyp70445-bib-0026] Kanthak, M. K. , T. Stalder , L. K. Hill , J. F. Thayer , M. Penz , and C. Kirschbaum . 2017. “Autonomic Dysregulation in Burnout and Depression: Evidence for the Central Role of Exhaustion.” Scandinavian Journal of Work, Environment & Health 43, no. 5: 475–484. 10.5271/sjweh.3647. PMC5788013 28514792

[psyp70445-bib-0027] Kemp, A. H. , D. S. Quintana , M. A. Gray , K. L. Felmingham , K. Brown , and J. M. Gatt . 2010. “Impact of Depression and Antidepressant Treatment on Heart Rate Variability: A Review and Meta‐Analysis.” Biological Psychiatry 67, no. 11: 1067–1074. 10.1016/j.biopsych.2009.12.012. 20138254

[psyp70445-bib-0028] Kleiger, R. E. , J. T. Bigger , M. S. Bosner , et al. 1991. “Stability Over Time of Variables Measuring Heart Rate Variability in Normal Subjects.” American Journal of Cardiology 68, no. 6: 626–630. 10.1016/0002-9149(91)90355-O. 1877480

[psyp70445-bib-0029] Kline, R. B. 2016. Principles and Practice of Structural Equation Modeling. 4th ed. Guilford Press.

[psyp70445-bib-0030] Koo, T. K. , and M. Y. Li . 2016. “A Guideline of Selecting and Reporting Intraclass Correlation Coefficients for Reliability Research.” Journal of Chiropractic Medicine 15, no. 2: 155–163. 10.1016/j.jcm.2016.02.012. PMC4913118 27330520

[psyp70445-bib-0031] Laborde, S. , E. Mosley , and J. F. Thayer . 2017. “Heart Rate Variability and Cardiac Vagal Tone in Psychophysiological Research—Recommendations for Experiment Planning, Data Analysis, and Data Reporting.” Frontiers in Psychology 8: 213. 10.3389/fpsyg.2017.00213. PMC5316555 28265249

[psyp70445-bib-0032] Lee, H. , H.‐L. Yang , H. G. Ryu , et al. 2023. “Real‐Time Machine Learning Model to Predict In‐Hospital Cardiac Arrest Using Heart Rate Variability in ICU.” Npj Digital Medicine 6, no. 1: 215. 10.1038/s41746-023-00960-2. PMC10665411 37993540

[psyp70445-bib-0033] Lewis, G. F. , S. A. Furman , M. F. McCool , and S. W. Porges . 2012. “Statistical Strategies to Quantify Respiratory Sinus Arrhythmia: Are Commonly Used Metrics Equivalent?” Biological Psychology 89, no. 2: 349–364. 10.1016/j.biopsycho.2011.11.009. PMC3269511 22138367

[psyp70445-bib-0034] Li, Z. , H. Snieder , S. Su , et al. 2009. “A Longitudinal Study in Youth of Heart Rate Variability at Rest and in Response to Stress.” International Journal of Psychophysiology 73, no. 3: 212–217. 10.1016/j.ijpsycho.2009.03.002. PMC2719684 19285108

[psyp70445-bib-0035] Penz, M. , M. K. Wekenborg , L. Pieper , et al. 2018. “The Dresden Burnout Study: Protocol of a Prospective Cohort Study for the Bio‐Psychological Investigation of Burnout.” International Journal of Methods in Psychiatric Research 27, no. 2: e1613. 10.1002/mpr.1613. PMC6877172 29611872

[psyp70445-bib-0036] Pinna, G. D. , R. Maestri , A. Torunski , et al. 2007. “Heart Rate Variability Measures: A Fresh Look at Reliability.” Clinical Science 113, no. 3: 131–140. 10.1042/CS20070055. 17381425

[psyp70445-bib-0037] Pitzalis, M. V. , F. Mastropasqua , F. Massari , et al. 1996. “Short‐ and Long‐Term Reproducibility of Time and Frequency Domain Heart Rate Variability Measurements in Normal Subjects.” Cardiovascular Research 32, no. 2: 226–233. 10.1016/0008-6363(96)00086-7. 8796108

[psyp70445-bib-0038] R Core Team . 2024. R: A Language and Environment for Statistical Computing. R Foundation for Statistical Computing. https://www.R‐project.org/.

[psyp70445-bib-0039] Ramesh, A. , T. Nayak , M. Beestrum , G. Quer , and J. A. Pandit . 2023. “Heart Rate Variability in Psychiatric Disorders: A Systematic Review.” Neuropsychiatric Disease and Treatment 19: 2217–2239. 10.2147/NDT.S429592. PMC10596135 37881808

[psyp70445-bib-0040] Rosseel, Y. 2012. “Lavaan: An R Package for Structural Equation Modeling.” Journal of Statistical Software 48, no. 2: 1–36. 10.18637/jss.v048.i02.

[psyp70445-bib-0041] Salomon, K. 2005. “Respiratory Sinus Arrhythmia During Stress Predicts Resting Respiratory Sinus Arrhythmia 3 Years Later in a Pediatric Sample.” Health Psychology 24, no. 1: 68–76. 10.1037/0278-6133.24.1.68. 15631564

[psyp70445-bib-0042] Sandercock, G. R. , P. D. Bromley , and D. A. Brodie . 2005. “The Reliability of Short‐Term Measurements of Heart Rate Variability.” International Journal of Cardiology 103, no. 3: 238–247. 10.1016/j.ijcard.2004.09.013. 16098384

[psyp70445-bib-0043] Saul, J. P. 1990. “Beat‐To‐Beat Variations of Heart Rate Reflect Modulation of Cardiac Autonomic Outflow.” Physiology 5, no. 1: 32–37. 10.1152/physiologyonline.1990.5.1.32.

[psyp70445-bib-0044] Schroeder, E. B. , E. A. Whitsel , G. W. Evans , R. J. Prineas , L. E. Chambless , and G. Heiss . 2004. “Repeatability of Heart Rate Variability Measures.” Journal of Electrocardiology 37, no. 3: 163–172. 10.1016/j.jelectrocard.2004.04.004. 15286929

[psyp70445-bib-0045] Schumann, A. , F. Lukas , K. Rieger , Y. Gupta , and K.‐J. Bär . 2025. “One‐Week Test‐Retest Recordings of Resting Cardiorespiratory Data for Reliability Analysis.” Scientific Data 12, no. 1: 12. 10.1038/s41597-024-04303-y. PMC11698850 39754019

[psyp70445-bib-0046] Seidman, A. J. , L. M. Bylsma , X. Yang , J. R. Jennings , C. J. George , and M. Kovacs . 2024. “Long‐Term Stability of Respiratory Sinus Arrhythmia Among Adults With and Without a History of Depression.” Psychophysiology 61, no. 1: e14427. 10.1111/psyp.14427. PMC10872939 37646340

[psyp70445-bib-0047] Sinnreich, R. , J. D. Kark , Y. Friedlander , D. Sapoznikov , and M. H. Luria . 1998. “Five Minute Recordings of Heart Rate Variability for Population Studies: Repeatability and Age–Sex Characteristics.” Heart 80, no. 2: 156–162. 10.1136/hrt.80.2.156. PMC1728778 9813562

[psyp70445-bib-0048] Sloan, R. P. , P. A. Shapiro , E. Bagiella , J. M. Gorman , and J. T. Bigger Jr. 1995. “Temporal Stability of Heart Period Variability During a Resting Baseline and in Response to Psychological Challenge.” Psychophysiology 32, no. 2: 191–196. 10.1111/j.1469-8986.1995.tb03311.x. 7630984

[psyp70445-bib-0049] Steyer, R. , D. Ferring , and M. J. Schmitt . 1992. “States and Traits in Psychological Assessment.” European Journal of Psychological Assessment 8, no. 2: 79–98.

[psyp70445-bib-0050] Steyer, R. , A. Mayer , C. Geiser , and D. A. Cole . 2015. “A Theory of States and Traits—Revised.” Annual Review of Clinical Psychology 11: 71–98. 10.1146/annurev-clinpsy-032813-153719. 25062476

[psyp70445-bib-0051] Steyer, R. , and M. J. Schmitt . 1990. “Latent State‐Trait Models in Attitude Research.” Quality and Quantity 24, no. 4: 427–445. 10.1007/BF00152014.

[psyp70445-bib-0052] Task Force of the European Society of Cardiology and the North American Society of Pacing and Electrophysiology . 1996. “Heart Rate Variability: Standards of Measurement, Physiological Interpretation, and Clinical Use.” Circulation 93, no. 5: 1043–1065. 10.1161/01.CIR.93.5.1043. 8598068

[psyp70445-bib-0053] Thayer, J. F. , F. Ahs , M. Fredrikson , J. J. Sollers , and T. D. Wager . 2012. “A Meta‐Analysis of Heart Rate Variability and Neuroimaging Studies: Implications for Heart Rate Variability as a Marker of Stress and Health.” Neuroscience and Biobehavioral Reviews 36, no. 2: 747–756. 10.1016/j.neubiorev.2011.11.009. 22178086

[psyp70445-bib-0054] Thayer, J. F. , and J. Koenig . 2025. “The Neurobiology of Self‐Regulation: A Neurovisceral Integration Perspective.” In APA Handbook of Health Psychology, Volume 1: Foundations and Context of Health Psychology, vol. 1, 335–348. American Psychological Association. 10.1037/0000394-015.

[psyp70445-bib-0055] Thayer, J. F. , S. S. Yamamoto , and J. F. Brosschot . 2010. “The Relationship of Autonomic Imbalance, Heart Rate Variability and Cardiovascular Disease Risk Factors.” International Journal of Cardiology 141, no. 2: 122–131. 10.1016/j.ijcard.2009.09.543. 19910061

[psyp70445-bib-0056] Uhlig, S. , A. Meylan , and U. Rudolph . 2020. “Reliability of Short‐Term Measurements of Heart Rate Variability: Findings From a Longitudinal Study.” Biological Psychology 154: 107905. 10.1016/j.biopsycho.2020.107905. 32505705

[psyp70445-bib-0057] Watanabe, T. , Y. Sugiyama , Y. Sumi , et al. 2002. “Effects of Vital Exhaustion on Cardiac Autonomic Nervous Functions Assessed by Heart Rate Variability at Rest in Middle‐Aged Male Workers.” International Journal of Behavioral Medicine 9, no. 1: 68–75. 10.1207/S15327558IJBM0901_05. 12112997

[psyp70445-bib-0058] Weckesser, L. J. , F. Plessow , M. Pilhatsch , M. Muehlhan , C. Kirschbaum , and R. Miller . 2014. “Do Venepuncture Procedures Induce Cortisol Responses? A Review, Study, and Synthesis for Stress Research.” Psychoneuroendocrinology 46: 88–99. 10.1016/j.psyneuen.2014.04.012. 24882161

[psyp70445-bib-0059] Wekenborg, M. K. , C. Rominger , and A. R. Schwerdtfeger . 2025. “Association Between Phasic Vagal‐Mediated Heart Rate Variability and Momentary Exhaustion in Daily Life.” Stress and Health 41, no. 4: e70074. 10.1002/smi.70074. PMC12282498 40693838

[psyp70445-bib-0060] Wekenborg, M. K. , A. Schwerdtfeger , N. Rothe , et al. 2022. “Determining the Direction of Prediction of the Association Between Parasympathetic Dysregulation and Exhaustion Symptoms.” Scientific Reports 12, no. 1: 10648. 10.1038/s41598-022-14743-4. PMC9219378 35739224

[psyp70445-bib-0061] Williams, D. P. , J. Koenig , L. Carnevali , et al. 2019. “Heart Rate Variability and Inflammation: A Meta‐Analysis of Human Studies.” Brain, Behavior, and Immunity 80: 219–226. 10.1016/j.bbi.2019.03.009. 30872091

[psyp70445-bib-0062] Wittling, W. 2014. Verfahren und Vorrichtung zur Bestimmung der Herzfrequenzvariabilität eines Lebewesens (Europäisches Patent Nr. EP 2 745 770 B1). European Patent Office.

